# Trapped-ion based nanoscale quantum sensing

**DOI:** 10.1186/s40580-025-00479-0

**Published:** 2025-02-21

**Authors:** Jieun Yoo, Hyunsoo Kim, Hyerin Kim, Yeongseo Kim, Taeyoung Choi

**Affiliations:** Department of Physics, Ewha Womans University, Seoul, 03760 Republic of Korea

**Keywords:** Quantum sensing, Trapped-ion, Magnetometry, Electrometry

## Abstract

Recent development of controlling quantum systems has enabled us to utilize the systems for quantum computing, communication, and sensing. In particular, quantum sensing has attracted attention to a broad community of science and technology, as it could surpass classical limitations in measuring physical quantities such as electric and magnetic field with unprecedented precision. Among various physical platforms for quantum sensing, trapped-ion based system possesses several advantages—atomic size, outstanding quantum coherence, and quantum properties. In this review, we introduce previous research efforts to utilize the trapped-ion system for reaching ultimate sensitivity and discuss future perspective and research directions in this emerging field.

**Graphical Abstract:**

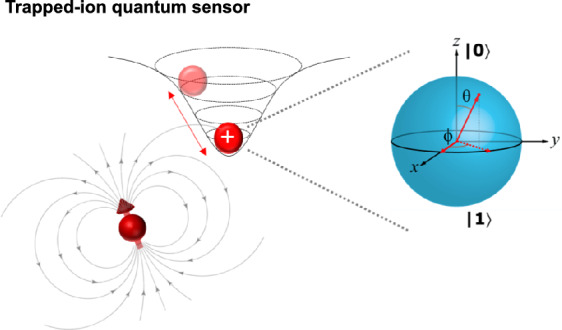

## Introduction

Sensing magnetic and electric fields, along with the associated forces, with improved spatial and energy resolution has always been paid attention to the field of physics, chemistry, material science, and biomedical sciences since such sensing techniques can be used for investigating spin physics, revealing unknown chemical structures, engineering electronic and magnetic devices, and scanning biological objects via resonance techniques. On the other hand, there has been tremendous development in controlling and utilizing quantum systems toward quantum computing, communication, and sensing [1–3]. In particular, achieving both nanoscale spatial resolution and quantum-limited sensitivity for probing fields and forces have been one of highly challenging research goals. Various physical platforms, such as superconducting quantum interference devices (SQUID) [4–8], vapor cells [9–15], Bose–Einstein condensates [16, 17], trapped-ions [18–24], solid-state spins (e.g., nitrogen-vacancy center in diamond) [25–30] and Rydberg atoms [31, 32] have been utilized to develop quantum sensors toward these goals, as summarized in Table 1.

**Table 1:** Comparisons of state-of-art quantum sensors

Comparisons of state-of-the-art quantum sensing					
Type	Measured quantity(ies)	Sensitivity ($/\sqrt{\text{Hz}}$)	Frequency range	Length scale	Reference
Vapor cell	Magnetic field	1pT to 160aT	40Hz-200Hz	~ 100um	[9–15]
SQUID	Magnetic field	0.08fT	Above 100Hz	~ 5um	[4–8]
BEC-ensemble	Magnetic field	8.3pT	DC and AC	~ 1um	[16, 17]
NV center in diamond	Magnetic field	0.9pT	1.5MHz	~ 5nm	[25–28]
	Electric field	202V/cm	AC		[29]
Si-V center	Magnetic field	100nT	DC and AC	~ 1um	[30]
Trapped ion(s)	Magnetic field	4.6pT	DC and AC	~ 1um	[18–21]
	Electric field	220nV/m	AC		[22–24]
Rydberg atom	Electric field / force	300nV/m	DC and AC	~ 1um	[31, 32]

Among these quantum platforms, trapped-ion systems are particularly advantageous due to their atomic size, long quantum coherence times, and high fidelity for state initialization, detection, and quantum gate operations [33, 34]. Leveraging these pristine quantum properties, substantial research efforts have focused on developing trapped-ion platforms as quantum sensors. This review presents key advancements and achievements in trapped-ion based quantum sensing of magnetic and electric fields, as well as relevant forces, while exploring the perspectives and potential of this expanding field.

## Trapped-ion based magnetic field/force sensing: methodologies and techniques

**Fig. 1:**
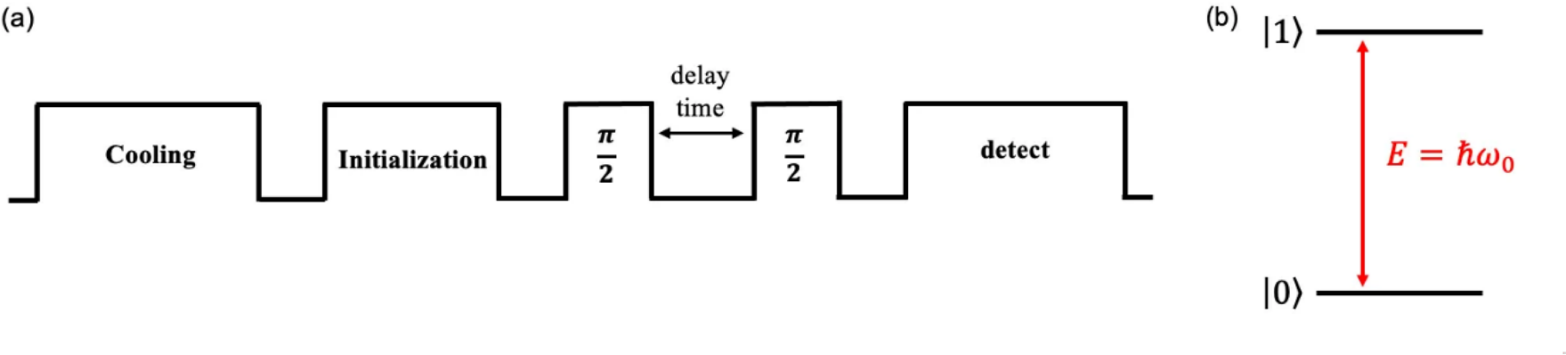
A schematic of a trapped-ion based quantum sensing protocol. **a** Ramsey spectroscopy in a trapped-ion system. **b** A two-level quantum system, where $\left|0\rangle \right.$ and $\left|1\rangle \right.$ represent the lower and higher energy state, respectively. ${{\varvec{\omega}}}_{0}$ is the transition frequency between these two levels

The basic principle of quantum magnetometry involves mapping static (DC) and time-varying (AC) magnetic fields onto the quantum phase accumulation of qubits and probing the phase with precise quantum control. In trapped-ion based magnetic sensors, atomic Zeeman levels are employed because these energy levels shift linearly in response to changes in the external magnetic field. Ramsey and Rabi spectroscopy on the Zeeman levels serve as primary experimental techniques for measuring these energy shifts and corresponding phase changes [1], as illustrated in Fig. 1. First, the states of the trapped ions are initialized to the $\left|0\rangle \right.$ state. A $\pi /2$ pulse is then applied to the qubit, resulting in a superposition state as $\left|\psi (t=0)\rangle \right.=\frac{1}{\sqrt{2}}\left( \left|0\rangle \right.+\left|1\rangle \right.\right)$ and this state evolves to $\left|\psi \left(t\right)\rangle \right.=\frac{1}{\sqrt{2}}\left( \left|0\rangle \right.+{e}^{-i{\omega }_{0}t}\left|1\rangle \right.\right)$ under the Hamiltonian given by $\widehat{H}=\hslash {\omega }_{0}{\widehat{\sigma }}_{z}$, where the qubit accumulates a phase of $\phi ={\omega }_{0}t$. Subsequently, a second $\pi /2$ pulse is applied to the qubit to project the accumulated phase onto a measurable state. The measured population of $\left|1\rangle \right.$ state can then be expressed as follows.

1 $$P_{{\left| {1\rangle } \right.}} = \frac{1}{2}\left( {1 - \cos \left( {\omega_{0} t} \right)} \right)$$

When an external magnetic field is applied to the qubit, its energy levels shift accordingly, enabling direct measurement of the change in ${\omega }_{0}$ to sense the magnetic field. Another basic approach for magnetic field sensing is to use the Rabi measurement. Similar to the Ramsey measurement, this process begins with an initialization pulse that prepares all states to the $\left|0\rangle \right.$ state. A resonant pulse is then applied to the qubit over a time increment, allowing measurement of the transition rate (Ω) between $\left|0\rangle \right.$ and $\left|1\rangle \right.$, known as the Rabi rate. In this case, the measured population of the $\left|1\rangle \right.$ state can be expressed as below.

2 $${P}_{\left|1\rangle \right.}=\frac{{\Omega }^{2}}{{\Omega }^{2}+{{\omega }_{0}}^{2}}{\text{sin}}^{2}(\frac{\sqrt{{\Omega }^{2}+{{\omega }_{0}}^{2}}}{2}t)$$

Therefore, the external magnetic field can influence ${\omega }_{0}$ and the measurement of ${P}_{\left|1\rangle \right.}$ as a function of $\Omega$ and $t$ enables us to extract the magnetic field information from the system. These two techniques–Ramsey and Rabi measurements–serve as basic methods for quantum sensing across various quantum platforms.

The performance of quantum magnetometry is primarily characterized by two key factors—sensitivity and spatial resolution. In general, sensitivity improves as the number of qubits increases, since more qubits enhance the signal-to-noise ratio during sensing. However, spatial resolution decreases as the number of qubits increases, since they occupy more physical dimensions. Thus, the mutually exclusive relationship between sensitivity and spatial resolution has been a critical consideration in the design of magnetometers for quantum sensing. In the following sections, we will discuss advanced techniques to optimize trapped-ion sensors for improving both sensitivity and spatial resolution within this relationship.

### Hahn-echo sequence

The sensitivity of a quantum magnetometer is proportional to both the number of qubits employed in sensing and the coherence time of these qubits as expressed in the equation below.

3 $${\left|\delta {B}_{s}\right|}_{min }\sim \frac{\hslash }{{\mu }_{B}\sqrt{n{T}_{measure}{T}_{2}}}$$

where $\hslash$ represents Planck’s constant divided by $2\pi$, ${\mu }_{B}$ is the Bohr magneton, $n$ is the number of qubits, ${T}_{measure}$ is the total sensing time, and ${T}_{2}$ are the coherence time of the qubits. On the other hand, another key factor for the sensor is to enhance its responsivity to the signal of interest and to detect the target signal selectively while minimizing environmental noise, where this capability is quantified by the signal-to-noise ratio (SNR).

**Fig. 2:**
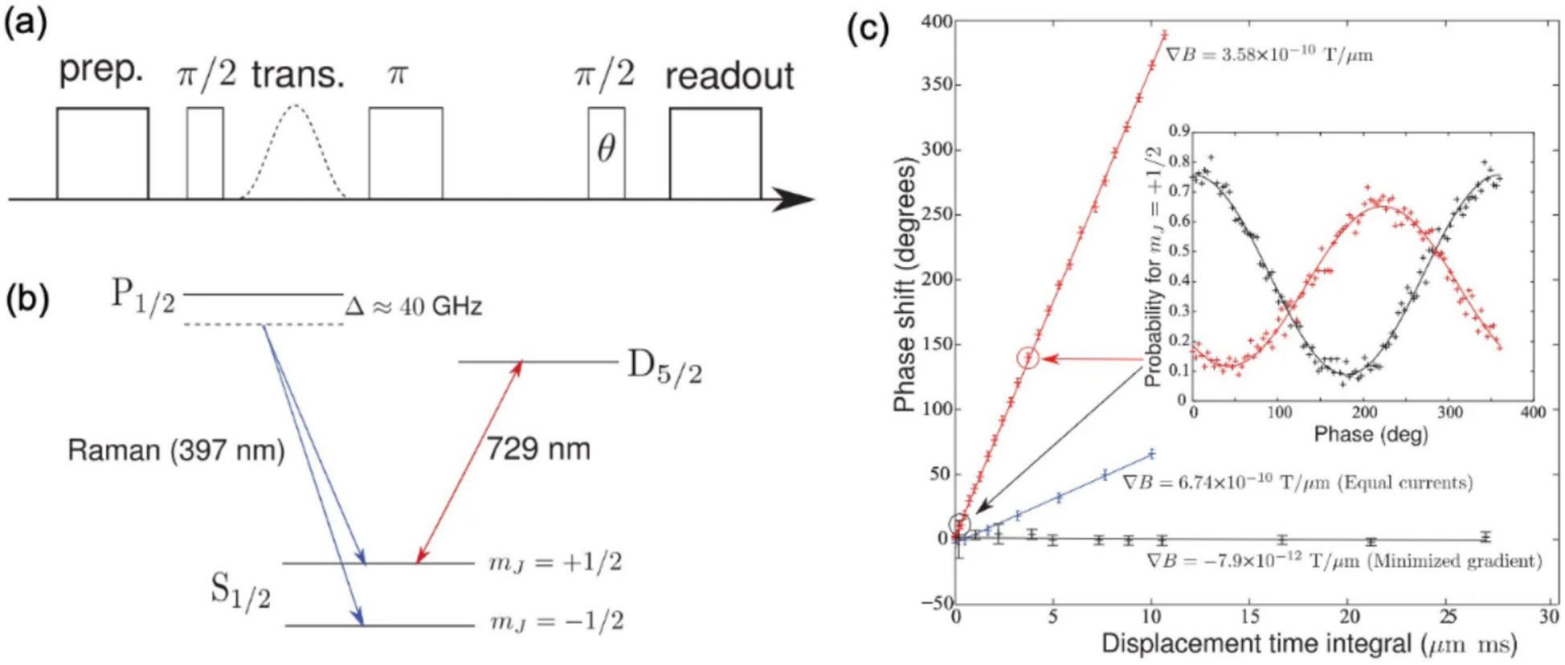
**a** Hahn-echo pulse sequence used to measure the magnetic field gradient. **b** Reduced level diagram of the ${}^{40}{\text{Ca}}^{+}$. **c** Recorded phase shift as a function of transported distance and time [Fig. 2 reprinted with permission from ref [35], Copyright (2016) American Physical Society.]

To improve the SNR, dynamical decoupling (DD) techniques are employed to reduce the coupling between the qubit and environmental noise, thus enhancing the coherence time of the qubit ${(T}_{2}$) and correcting unwanted phase shifts that occur during the sensing process. Figure 2a illustrates the schematic of a Hahn-echo sequence to correct phase shifts arising from dephasing. By applying a single $\pi$ pulse after the first $\pi /2$ pulse, the time-dependent phase shift can be fully reversed, allowing the dephased signal to be recollected. A second $\pi /2$ pulse is then applied for state detection. As a result, this Hahn-echo sequence effectively extends the coherence time of the qubit ${(T}_{2}$), enhancing sensitivity (as indicated in Eq. 3) and reducing the dephasing noise during the sensing process, thereby maximizing the signal of interest.

Since the trapped-ion system possesses a long coherence time compared to other sensing platforms [36], it is possible to shuttle the atom-size sensor to regions of interest and measure both the local magnetic field and its gradient while maintaining coherence [35]. Figure 2 shows the Hahn-echo measurement of the magnetic field gradient using the Zeeman levels of ${}^{40}{\text{Ca}}^{+}$ (Fig. 2b). In this experiment, the superposition state of two Zeeman levels is prepared using the first $\pi /2$ pulse. The ion is then shuttled a distance on the order of 100 μm across the trapping zone (indicated by the dotted Gaussian envelope in Fig. 2a), returned to its original position, and the remaining Hahn-echo sequence is performed. During the shuttling process, the ion senses local magnetic fields, which may induce off-resonant transitions between the two Zeeman levels. When magnetic field remains constant over time, this phase shift can be expressed as a function of the ion's position and the magnetic field gradient, as shown below.

4 $${\phi }_{rad}= \frac{g{\mu }_{B}}{\hslash }\frac{\delta B}{\delta x}{\int }_{0}^{T}\left[{x}_{ion}(t)-{x}_{ion}(0)\right]dt$$

where $g$ is the Landé factor, and this equation (Eq. 4) is derived from $\phi ={\omega }_{0}t=\frac{g{\mu }_{B}B}{\hslash }t$. Figure 2(c) indicates the relationship between the ion’s displacement and its corresponding phase shift. Therefore, one can extract the magnetic field gradient and reduce it to less than ${10}^{-12} \text{T}/\mu \text{m}$. This technique can be extended to sense magnetic field gradients at nanometer scale in the femto-Tesla regime and to lengthen the coherence time of Zeeman qubits by minimizing the inhomogeneity of the magnetic field.

### Dynamic decoupling

**Fig. 3:**
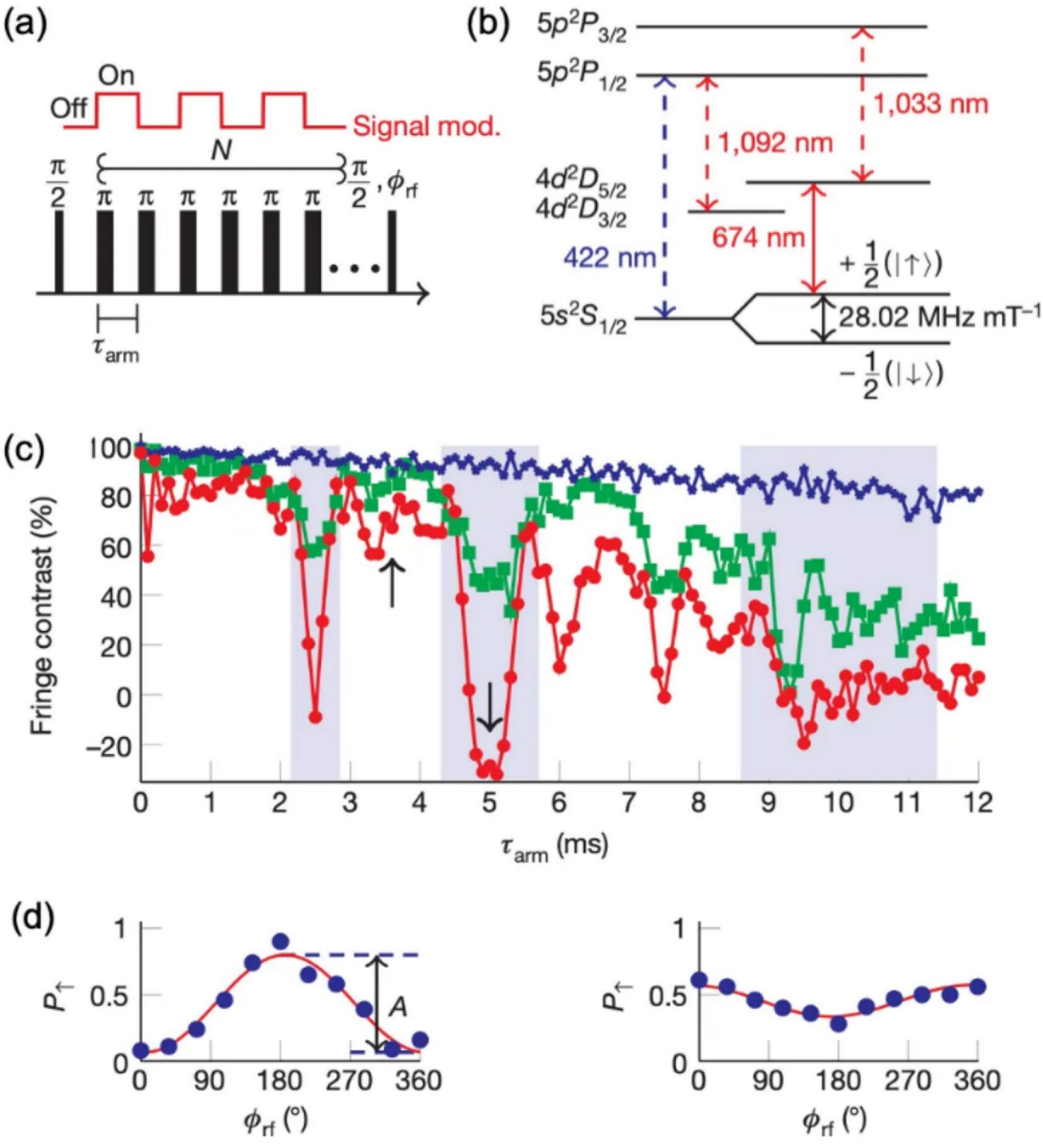
**a** A schematic of Quantum lock-in measurement pulses. **b** Level diagram of a ${}^{88}{\text{Sr}}^{+}$ ion. **c** Fringe contrast versus half-lock in period, ${\tau}_{a r m}$, data corresponding to N = 1, 9 and 17 pulses are shown using blue stars, green rectangles and red circles. (d) Probability of finding the ion in the $\left|\uparrow \rangle \right.$ state versus ${\phi}_{r f}$, solid line is a best fit to $P_{\uparrow} = \frac{1}{2} + \left( \frac{A}{2} \right) \cos \left( \phi_{rf} \right)$. [Fig. 3 reprinted with permission from ref [20], Copyright (2011) Nature]

While the Hahn-echo sequence is effective at mitigating broadband noises, one can further decouple time-dependent noise and enhance the desired signal from the sensing process by incorporating periodic $\pi$ pulses into the Hahn-echo sequence. This dynamic decoupling utilizes $N$ periodic $\pi$ pulses positioned at equal time intervals between two $\pi /2$ pulses. These repetitive $\pi$ pulses can be considered as external modulation with a frequency of ${f}_{m}$ (as illustrated in the Fig. 3a), and ${f}_{m}$ can be selected to be outside the noise bandwidth obtained from noise spectral density measurements [20]. This method is known as Lock-in technique, which significantly enhances the signal-to-noise ratio (SNR). When the modulation is added to the Zeeman qubits, the Hamiltonian can be written as follows.

5 $$H=\frac{M\left(t\right){\widehat{\sigma }}_{z}+\Omega \left(t\right){\widehat{\sigma }}_{x}}{2}$$

where ${\widehat{\sigma }}_{z}$ defines the Zeeman levels, $M\left(t\right)$ represents the magnetic signal, including external noise, ${\widehat{\sigma }}_{x}$ modulates the Zeeman levels, and $\Omega \left(t\right)$ denotes the amplitude of the synchronized modulation signal. During the repetitive $\pi$ pulses, the phase accumulation from the external noise decays, while the phase from the magnetic signal adds up coherently. The accumulated phase (${\phi }_{lock-in}$) at the superposition state, which is in principle the same as Eq. 5, is shown below.

6 $$\phi _{{lock - in}} = \frac{{g\mu _{B} }}{\hbar }\int_{0}^{T} {dtB\left( t \right){\text{cos}}\left( {\int_{0}^{t} {\Omega \left( {t^{\prime } } \right)dt^{\prime } } } \right)}$$

where $T$ is the total measurement time and $B\left(t\right)$ is the magnetic field.

In this experiment, the first $\pi /2$ pulse is applied to a ${}^{88}{\text{Sr}}^{+}$ ion qubit, creating a superposition state $\left|{\psi }_{0}\rangle \right.=\frac{1}{\sqrt{2}}\left( \left|\uparrow \rangle \right.+\left|\downarrow \rangle \right.\right)$ (Fig. 3b). Subsequently, $N$ periodic $\pi$ pulses with a time interval ${\tau }_{arm}$ modulate the total measured signal at a frequency of ${f}_{m}=1/(2{\tau }_{arm})$. As discussed in Sect. 2.1, the local magnetic field induces off-resonant transitions between the two Zeeman levels of the ion qubit, causing a phase shift (${\phi }_{lock-in}$) of the ion qubit. This phase shift can be extracted by measuring the probability of finding the qubit in the $\left|\uparrow \rangle \right.$ state (${P}_{\uparrow }$) as a function of the relative phase of the second $\pi /2$ pulse (${\phi }_{rf}$).

7 $${P}_{\uparrow }=\frac{1}{2}+\left(\frac{A}{2}\right)\text{cos}\left({\phi }_{rf}-{\phi }_{lock-in}\right)$$

where $A$ is the Ramsey fringe contrast. Figure 3c shows the fringe contrast ($A$) while varying the modulation frequency ${f}_{m}=1/(2{\tau }_{arm})$ and Fig. 3d indicates the phase scans at two arrows indicated in Fig. 3c. Thus, one can calculate the sensitivity of this Lock-in method based on the extracted values of ${\phi }_{lock-in}$ and $A$ as shown below.

8 $$s=\frac{1}{2\pi }\sqrt{\frac{4-{A}^{2}}{2{A}^{2}\left(N+1\right){\tau }_{arm}}}\text{Hz}/\sqrt{\text{Hz}}$$

where $A$ is the Ramsey fringe contrast, $N$ is the number of periodic pulses, and ${T}_{arm}$ is the time interval.

**Fig. 4:**
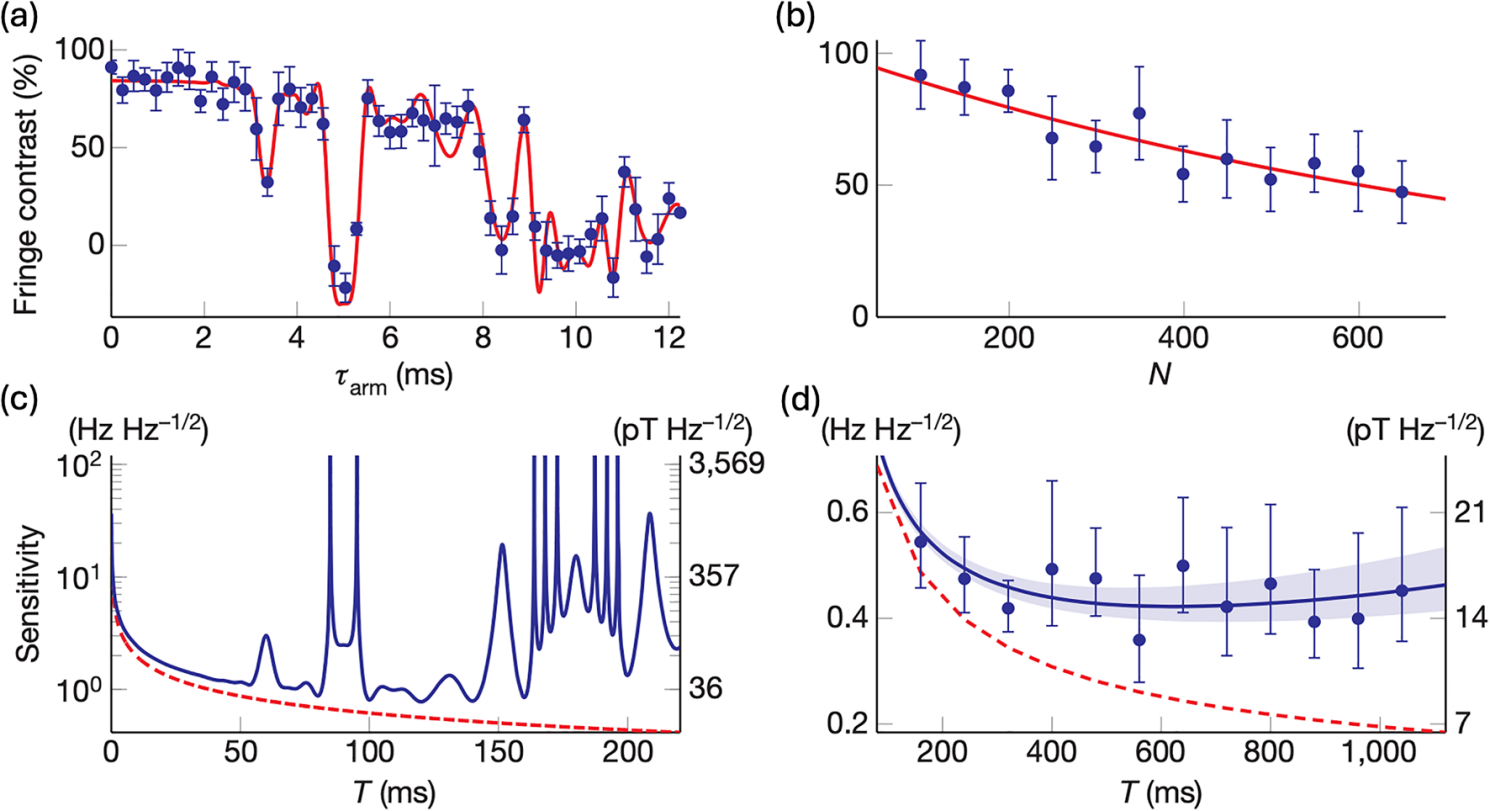
Sensitivity of the quantum lock-in measurement. **a** Fringe contrast versus ${\tau}_{a r m}$ for ${N} = 17 \pi$ pulses, red line is a best fit to calculated fringe contrast. **b** Fringe contrast versus number of $\pi$ pulses, $N$. **c** Lock-in sensitivity versus the lock-in sequence duration, $T$. **d** Exponential decay fit shown in (**b**), translated to sensitivity, dashed red line shows the standard quantum limit on the lock-in sensitivity. [Fig. 4 reprinted with permission from ref [20], Copyright (2011) Nature.]

Figure 4a and c display the fringe contrast and the extracted sensitivity for $N=17 \pi$ pulses, respectively. Increasing the number of $\pi$ pulses is advantageous for enhancing sensitivity, provided that the coherence time of the qubit permits it. The frequency sensitivity can be converted to magnetic field sensitivity using the Zeeman energy-frequency relation ${\omega }_{0}=2\pi f=\frac{g{\mu }_{B}B}{\hslash }$. As shown in the Fig. 4b and d, by optimizing the number and the length of the modulation pulses, the trapped-ion sensor could achieve a magnetic field sensitivity of approximately 15 $\text{pT}/\sqrt{\text{Hz}}$, approaching the quantum limit indicated by the dashed red line in Fig. 4c and d).

### Dressed state

**Fig. 5:**
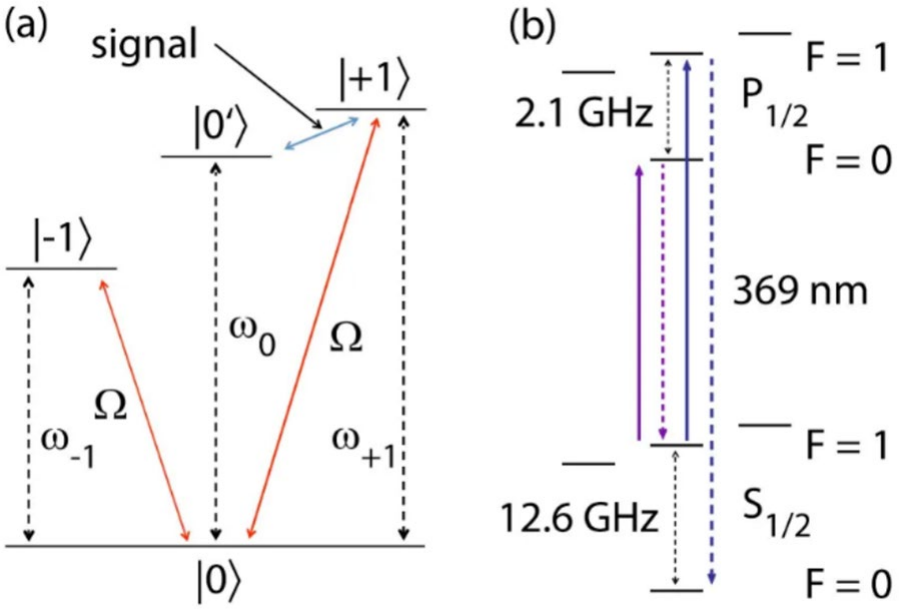
**a** Possible transitions driven by microwave pulses. **b** Energy level diagram of the ${}^{171}{\text{Yb}}^{+}$ qubit. [Fig. 5 reprinted with permission from ref [18], Copyright (2016) American Physical Society.]

**Fig. 6:**
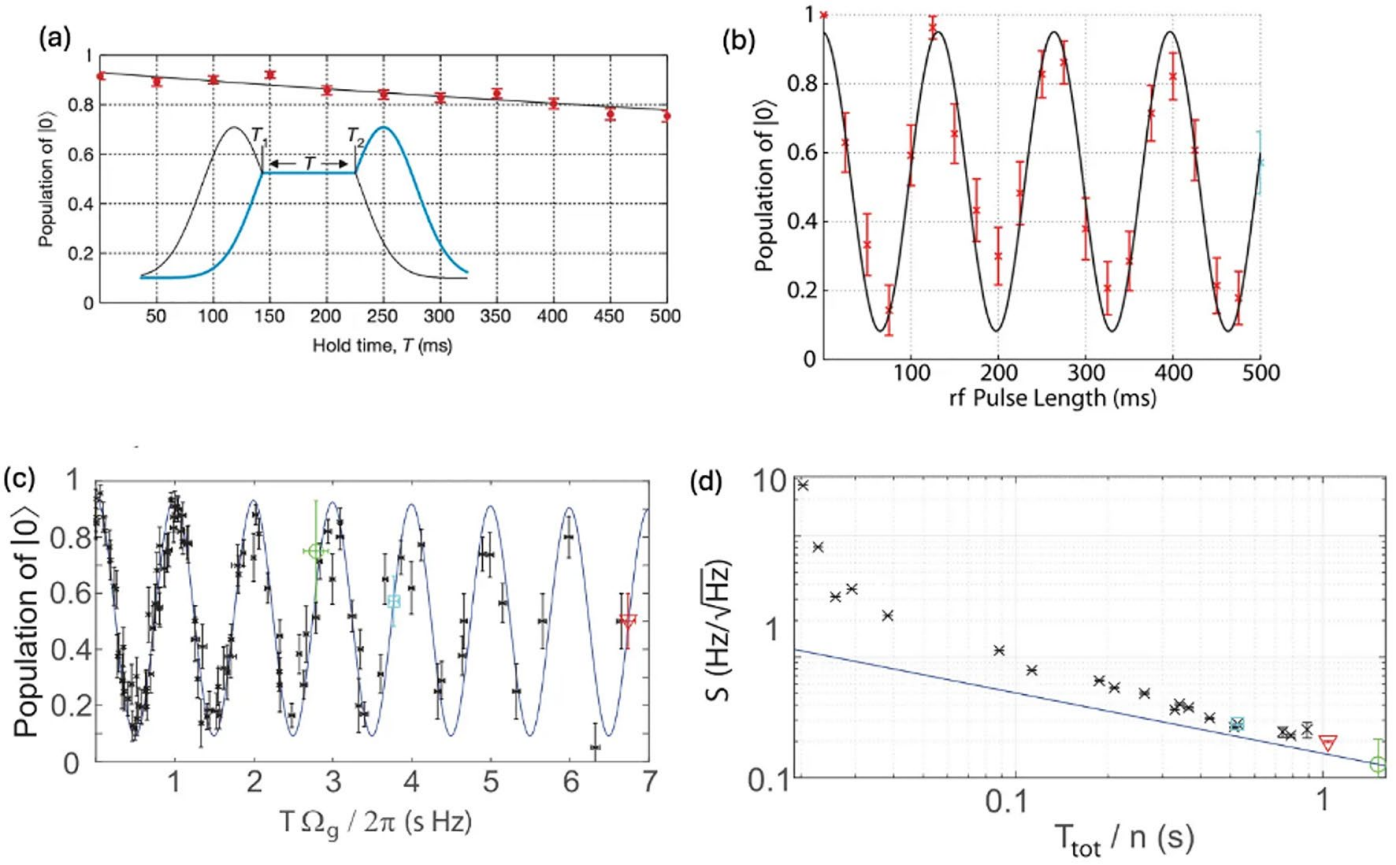
**a** Lifetime of the dressed state $\left| D \rangle \right.$ with the inset showing the STIRAP pulse sequence. **b** Coherence Rabi oscillation of the transition $\left|+1\rangle \right.$ to $\left|{0}^{\boldsymbol{^{\prime}}}\rangle \right.$. **c** Rabi oscillation measured over various durations $T$. **d** Sensitivity $S$ for variation in $\Omega_{g}$ as a function of the total measurement time $T_{t o t} / n$ (assuming $T_{t o t}$ $\sim$ $T$ when $T$ is significantly longer than any additional required time). [Fig. 6 **a** reprinted with permission from ref [37], Copyright (2011) Nature, Fig. 6 **b**–**d** reprinted with permission from ref [18], Copyright (2016) American Physical Society]

Zeeman-based qubit often exhibit limited coherence time due to their sensitivity to external magnetic field fluctuations, with typical coherence times on the order of several milliseconds. To address this limitation and extend the coherence time, dressed states can be utilized. These states are less sensitive to magnetic field noise and can increase coherence times by up to three orders of magnitude [18, 37]. Dressed states can be created via a stimulated Raman adiabatic passage (STIRAP) pulse [38]. In this approach, two Gaussian-enveloped microwave pulses are applied to the qubit, driving the transition from $\left|0\rangle \right.$ to $\left|+1\rangle \right.$ and from $\left|0\rangle \right.$ to $\left|-1\rangle \right.$, with a temporal shift between the two pulses (Fig. 5a and inset of Fig. 6a). This process results in an adiabatic transfer of the population from $\left|+1\rangle \right.$ to $\left|-1\rangle \right.$. The two pulses correspond to the left and right Gaussian envelopes in the inset of Fig. 6a.

Then, the dressed superposition states of $\left|B\rangle \right.=\left(\left|-1\rangle +\left|+1\rangle \right. \right.\right)/\sqrt{2}$ or $\left|D\rangle \right.=\left(\left|-1\rangle -\left|+1\rangle \right. \right.\right)/\sqrt{2}$ can be generated by precise control of the phase and timing on microwave pulses during this process. Coherent Rabi oscillation between the dressed state $\left|B\rangle \right.$ and the $\left|{0}{\prime}\rangle \right.$ state can then be induced by applying an additional radio frequency pulse at a frequency resonant with $\left|+1\rangle \right.$ to $\left|{0}{\prime}\rangle \right.$ transition for the measurement time $T$ (Fig. 6b). In this scheme, the qubit which is defined by the two states $\left|B\rangle \right.$ and $\left|{0}{\prime}\rangle \right.$ becomes robust against external magnetic field noise, achieving coherence times that extend to several seconds as shown in Fig. 6a [2, 23]. For magnetic field sensing, coherent transitions between $\left|+1\rangle \right.$ to $\left|{0}{\prime}\rangle \right.$ are driven with the Rabi rate ${\Omega }_{g}$ during the measurement time $T$. Variations in the external magnetic field causes fluctuation in ${\Omega }_{g}$ for the $\left|+1\rangle \right.$ to $\left|{0}{\prime}\rangle \right.$ transition, resulting in phase accumulation during the measurement time $T$ (inset of Fig. 6a). At the end end of the STIRAP sequence, the population of the $\left|B\rangle \right.$ state is transferred to the $\left|-1\rangle \right.$ state. Subsequently, an additional microwave $\pi$-pulse is applied to drive the transition from $\left|-1\rangle \right.$ to $\left|0\rangle \right.$, mapping the $\left|B\rangle \right.$ state onto $\left|0\rangle \right.$. Thus, the population in the $\left|0\rangle \right.$ state reflects the phase accumulated due to the external magnetic field.

Figure 6c presents the Rabi rate measurement as a function of the phase $\phi =T{\Omega }_{g}$, indicating that the accumulated phase is correlated with the population of $\left|0\rangle \right.$. The minimal detectable change in ${\Omega }_{g}$ from this measurement is determined by the minimal detectable population and the duration of the measurement as shown below.

9 $$\delta {\Omega }_{g}=\frac{\Delta P}{\left|\frac{\partial P(\phi )}{\partial \phi }\right|T}$$

where $\Delta P$, $\phi$, and $T$ are the standard deviation of population, the accumulated phase ($\phi ={\Omega }_{g}T$), and the measurement duration, respectively. The population $P$ of the $\left|0\rangle \right.$ state can be measured across different sets of ${\Omega }_{g}$ and $T$, thus providing a $P(\phi )$ plot as shown in Fig. 6c. Furthermore, the shot-noise-limited sensitivity $S$ for the ${\Omega }_{g}$ measurement is given by the Eq. (10) [39, 40]. This indicates that the sensitivity improves as the slope of $P(\phi )$ becomes steeper.

10 $$S \sim \delta {\Omega }_{g}\sqrt{T}$$

Figure 6d shows the sensitivity values extracted from the ${\Omega }_{g}$ measurement based on the Eq. 10. Three points, indicating a high slope of $P(\phi )$, are highlighted with different colors and symbols in Fig. 6c and d. For example, the best sensitivity in frequency using this dressed state scheme has been reported as 0.130 $\pm$ 0.036 $\text{Hz}/\sqrt{\text{Hz}}$ (represented by the green circle symbol in Fig. 6d). In addition, when the Zeeman energy of ${}^{171}{\text{Yb}}^{+}$ (Fig. 5b) is converted from the frequency to magnetic field, the magnetic field sensitivity reaches 4.6 $\text{pT}/\sqrt{\text{Hz}}$.

### Entanglement

Another approach to enhance the sensitivity of trapped-ion based quantum sensors is to utilize multi-ion qubit ensembles [21, 22]. According to Eq. 3, increasing the number of sensors—specifically, uncorrelated qubits—can improve measurement sensitivity. Alternatively, by employing highly correlated quantum states such as quantum entangled states, several advantages can be realized in quantum sensing. These advantages include more robust qubits with longer coherence times and the ability to perform remote sensing via entanglement, such as measuring magnetic field gradients through entangled ion qubit shuttling.

**Fig. 7:**
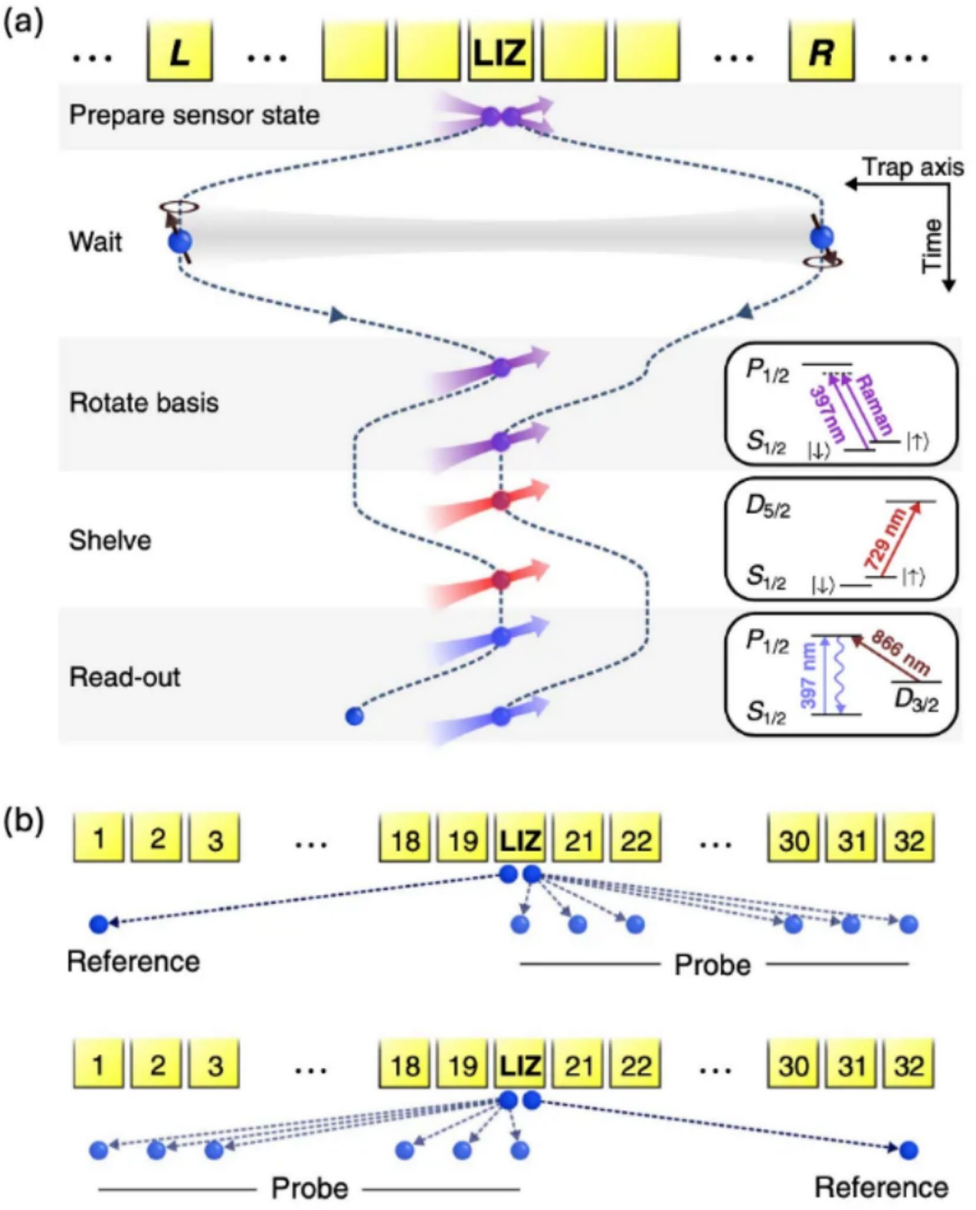
**a** Experimental procedure for measurements of inhomogeneous magnetic fields using ${}^{40}{\text{Ca}}^{+}$ ions. **b** A schematic representation of ion shuttling. [Fig. 7 reprinted with permission from ref [21], Copyright (2017) American Physical Society]

Figure 7a illustrates the experimental sequence designed to sense the magnetic field gradient using two entangled ion qubits in a Bell state, represented as ${\Psi }^{+}=\left(\left|\uparrow \downarrow \rangle +\left|\downarrow \uparrow \rangle \right.\right.\right)/\sqrt{2}$. In this experiment, each individual qubit utilizes two Zeeman ground states and the entangled state is created in the laser interaction zone (LIZ, indicated in Fig. 7) through applying a spin-dependent optical dipole force, such as the Mølmer–Sørensen gate.

After preparing the entangled state, the two entangled ions are separated and positioned away from the laser interaction zone (LIZ). In this configuration, one ion serves as a reference position while the other acts as a probe for sensing the magnetic field in the region of interest as shown in Fig. 7b. During the interrogation time $T$, the ions maintain their separate positions, allowing the phase accumulation of the entangled state. Following this interrogation time, the qubits are returned to the LIZ, where a basis rotation is executed to read out the state. The phase accumulation ${\varphi }_{dc}$ of the entangled state during the interrogation time is directly related to the difference in DC magnetic fields through the first-order Zeeman effect. This relationship can be expressed as follows.

11 $$\Delta \omega {\left({x}_{1}, {x}_{2}\right)}_{dc}\equiv {\dot{\varphi }}_{dc}=\frac{g{\mu }_{B}}{\hslash }\Delta B({x}_{1}, {x}_{2})$$

where the $g$ and ${\mu }_{B}$ are Landé factor and Bohr magneton, respectively.

**Fig. 8:**
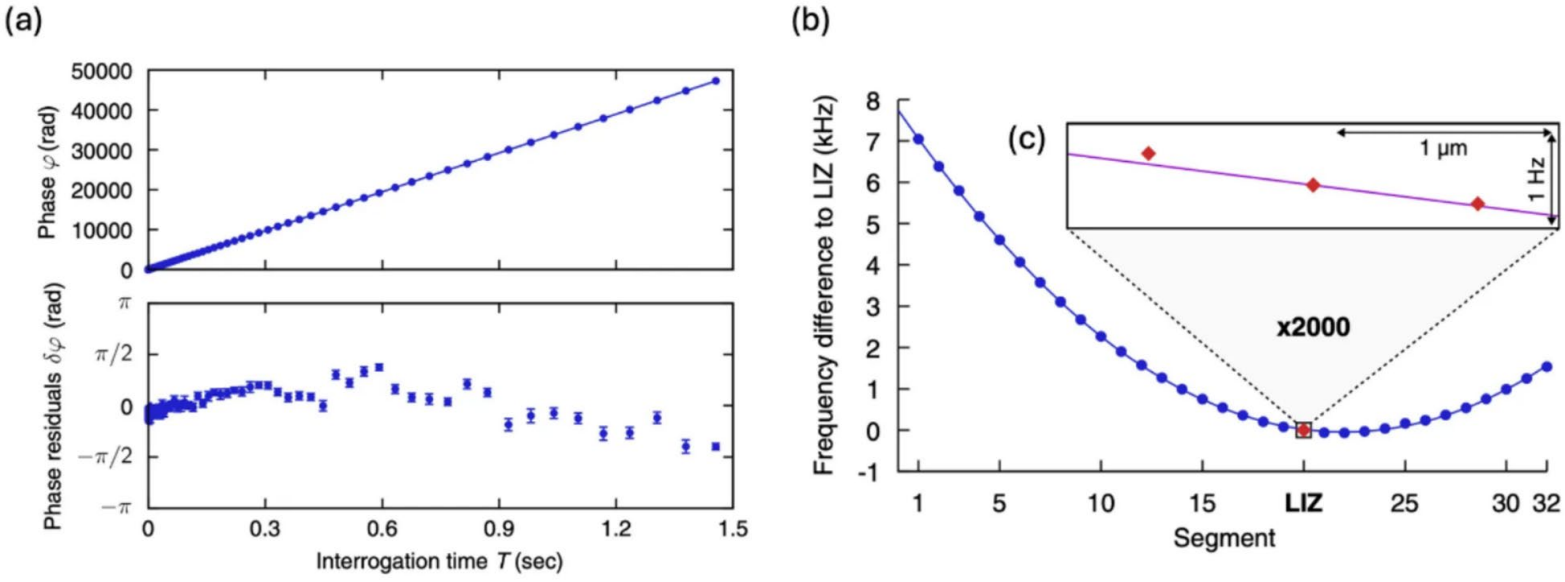
**a** Incremental measurement of the phase accumulation $\varphi_{d c}$ and its rate $\Delta \omega \left( = \dot{\varphi}_{d c} \right)$ at an ion distance of $d = 6.2 \, \text{mm}$. A linear fit to measurements of the accumulated phase $\boldsymbol{\varphi }$ at predefined interrogation times (top part), and the fit residuals $\delta \varphi$ for each phase measurement are shown (bottom part). **b** Frequency difference ($\Delta \omega$) from each segment. **c** Zoomed-in view of the position at the laser interaction zone (LIZ). [Fig. 8 reprinted with permission from ref [21], Copyright (2017) American Physical Society]

When two ions are shuttled and positioned at a fixed location, increasing the interrogation time $T$ leads to a phase shift that varies linearly with $T$, as shown in Fig. 8a. From this experimental data, we can extract the local DC magnetic fields and their standard deviations. Figure 8b depicts the qubit frequency difference over various ion positions and Fig. 8c provides a zoomed-in view of the position at the laser interaction zone (LIZ). Similar to the method employed in the dressed state scheme, the sensitivity of the frequency can be expressed as $S=\Delta {\upomega }_{DC}\sqrt{T}$, where $\Delta {\upomega }_{DC}$ is obtained by measuring the slope of the phase accumulation ${\varphi }_{dc}$ (note that $\Delta {\upomega }_{DC}$ replaces $\delta {\Omega }_{g}$ in the Eq. 10). By converting this frequency measurement to the corresponding Zeeman energy of ${}^{40}{\text{Ca}}^{+}$ ion, one can extract a sensitivity of approximately 12 $\text{pT}/\sqrt{\text{Hz}}$ with an accuracy of 300 $\text{fT}$. In particular, Fig. 8c indicates that the standard deviation for the ion position and frequency difference are around 10 $\text{nm}$ and 10 $\text{mH}z$ respectively. These findings suggest that ion qubits are indeed capable of functioning as highly sensitive nanoscale quantum sensors, making them valuable tools for precision measurements in various quantum sensing applications.

## Trapped-ion based electric field/force sensing: methodologies and techniques

In Chapter 2, we introduced the utilization of trapped ions for quantum sensing of magnetic fields and forces, exploring various techniques to enhance their sensitivity. These studies emphasized the modulation and accumulation of phase in pristine quantum states, such as superposition and entangled states, leading to improvements in magnetic field and frequency sensitivity. Furthermore, several studies have successfully demonstrated the sensing of electric fields and forces with sub-micrometer spatial resolution [22–24]. In this chapter, we aim to introduce these previous researches that highlight how the trapped-ion system can be also used to detect electric fields and forces with nanoscale spatial resolution, paving the way for a broader range of quantum sensing applications.

### Nanoscale imaging

**Fig. 9:**
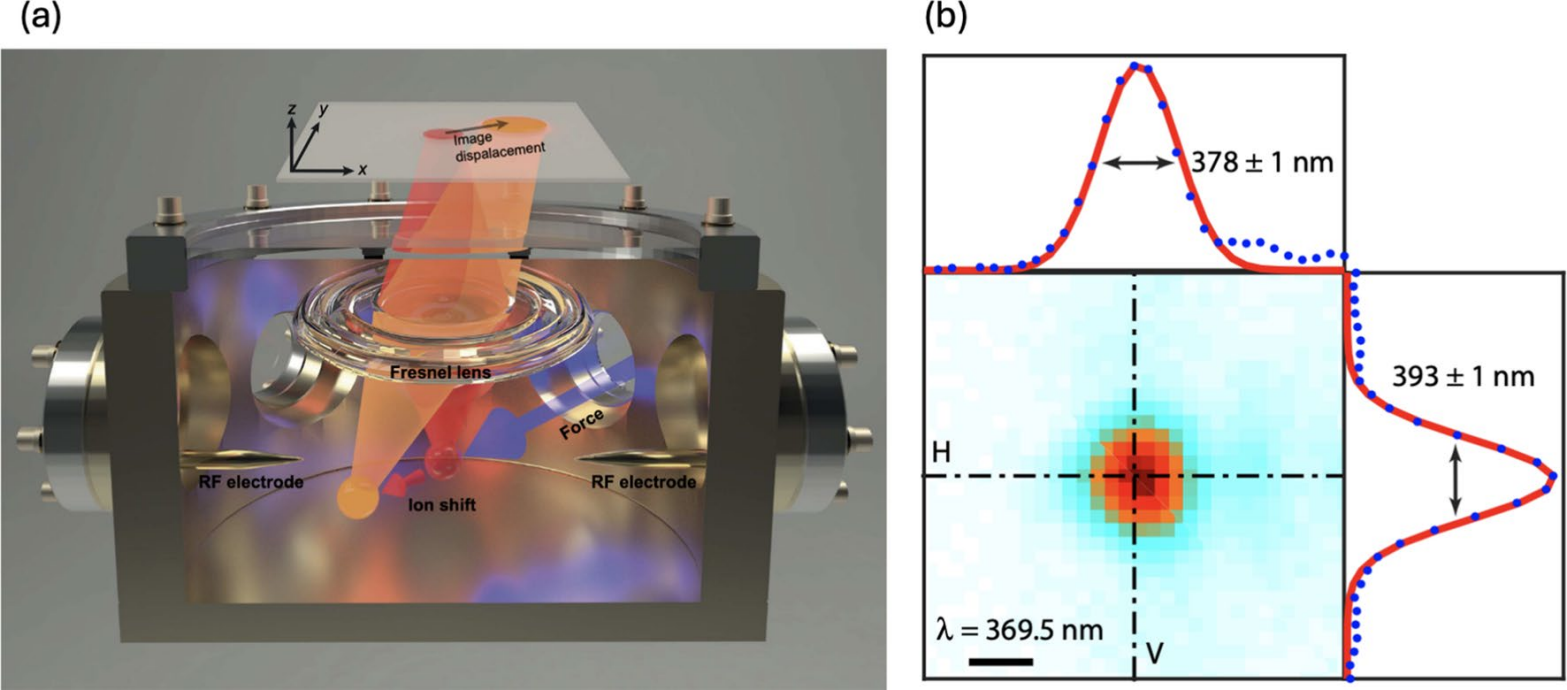
**a** Experimental setup for nanoscale force sensing using high resolution optics, **b** Wavelength scale ion image with Gaussian fitting, [Fig. 9 reprinted with permission from ref [24], Copyright (2018) Science AAAS]

One direct measure of spatial resolution in sensing is the precise determination of the positions of ion sensors. A straightforward approach involves analyzing fluorescence images of the ions during sensing or state detection. However, it is important to note that these optical images are diffraction-limited and may suffer from optical aberrations. In particular, high-resolution optics can be employed, such as a Fresnel lens, which help achieve the diffraction limit while minimizing optical aberrations as shown in Fig. 9b. By utilizing such advanced optical elements, the precision of ion imaging can be improved to reach resolution as fine as one nanometer [24].

**Fig. 10:**
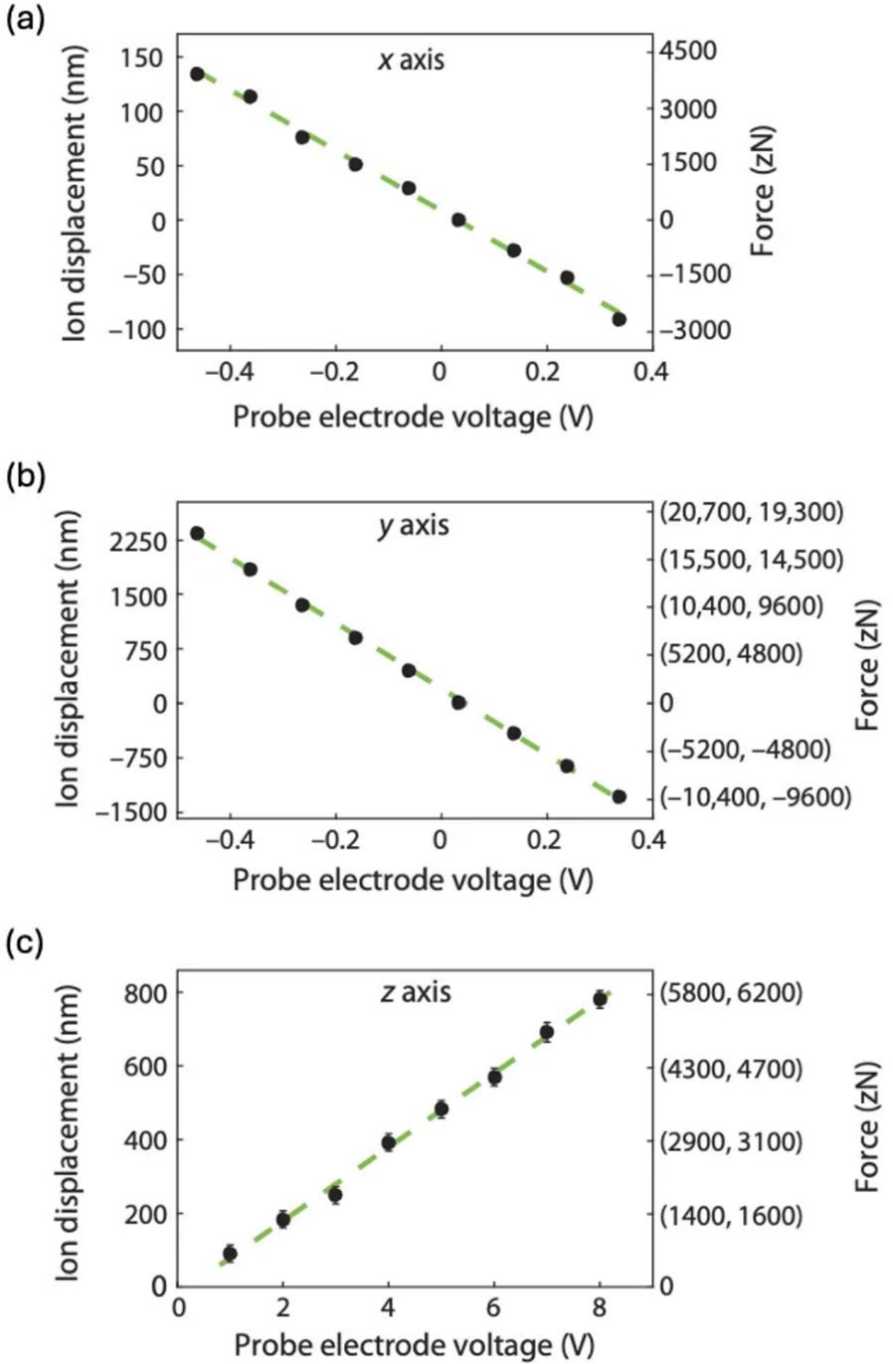
Electrostatic force detection and ion movement in three axis, x and y axes refer to camera plane, and z axis refers to the optical axis of the imaging system. **a** Ion displacement and applied electric force as a function of the applied voltage on electrodes in the x axis. **b** Same as (**a**), but for the y axis. **c** Same as (**b**), but for the z axis. [Fig. 10 reprinted with permission from ref [24], Copyright (2018) Science AAAS]

The ions are trapped under a time-averaged harmonic potential well, which is formed by fast saddle-shaped RF and DC potentials. The displacement of an ion can be mapped onto the force acting on the ion according to Hooke's law, expressed as ${F}_{i}={k}_{i}\Delta {x}_{i} ( {k}_{i}=m{\omega }_{i}^{2}, i=x, y, z)$. The time-averaged force in each direction is directly related to the ions’ trap frequencies $({\omega }_{i})$, which can be precisely measured using RF frequency-qubit fluorescence spectroscopy or qubit-motion spectroscopy in each direction. Thus, the spring constant ${k}_{i}(=m{\omega }_{i}^{2})$ can be extracted, and the $x, y, z$ displacements of the ion provide the magnitude of the force in each direction as shown in Fig. 10.

**Fig. 11:**
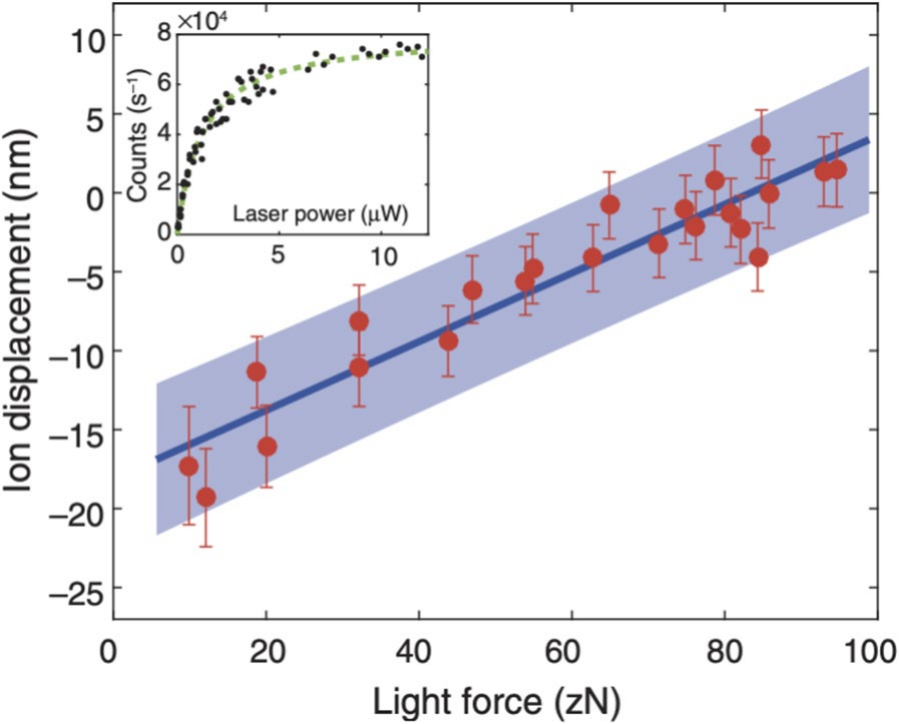
Light force detection using a single ion [Fig. 11 reprinted with permission from ref [24], Copyright (2018) Science AAAS]

Using this calibrated force–displacement relationship, one can measure the light force experienced by an ion due to ion-laser interaction. When a cooling laser beam is applied to the ion qubit, the resulting shift in the ions’ position can be directly measured using the Fresnel lens system (Fig. 9a), achieving position precision on the order of several nanometers. Based on this calibrated force–displacement relationship obtained in Fig. 10, the magnitude of the light force can be extracted, reaching down to zepto-Newton ($z\text{N}$) range, as depicted in Fig. 11. This work demonstrates the ability to measure the ions’ position down to the diffraction limit, achieving position precision at a nanometer spatial resolution through the use of ultra-high-resolution optics.

### Doppler velocimetry

When an external force is applied to the ions in the presence of resonant light, the ions will move relative to the light source, leading to changes in their time-averaged displacement (Chapter 3.1) or altering the frequency of the light from the ions' frame of reference. In the second case, the number of photons emitted from the ions will vary depending on the magnitude and direction of the external force. This technique is referred to as Doppler velocimetry. Here, we present a study that employs hundreds of trapped ions to measure forces down to several hundred yocto-Newtons with a spatial resolution of approximately 10 $\text{nm}$.

**Fig. 12:**
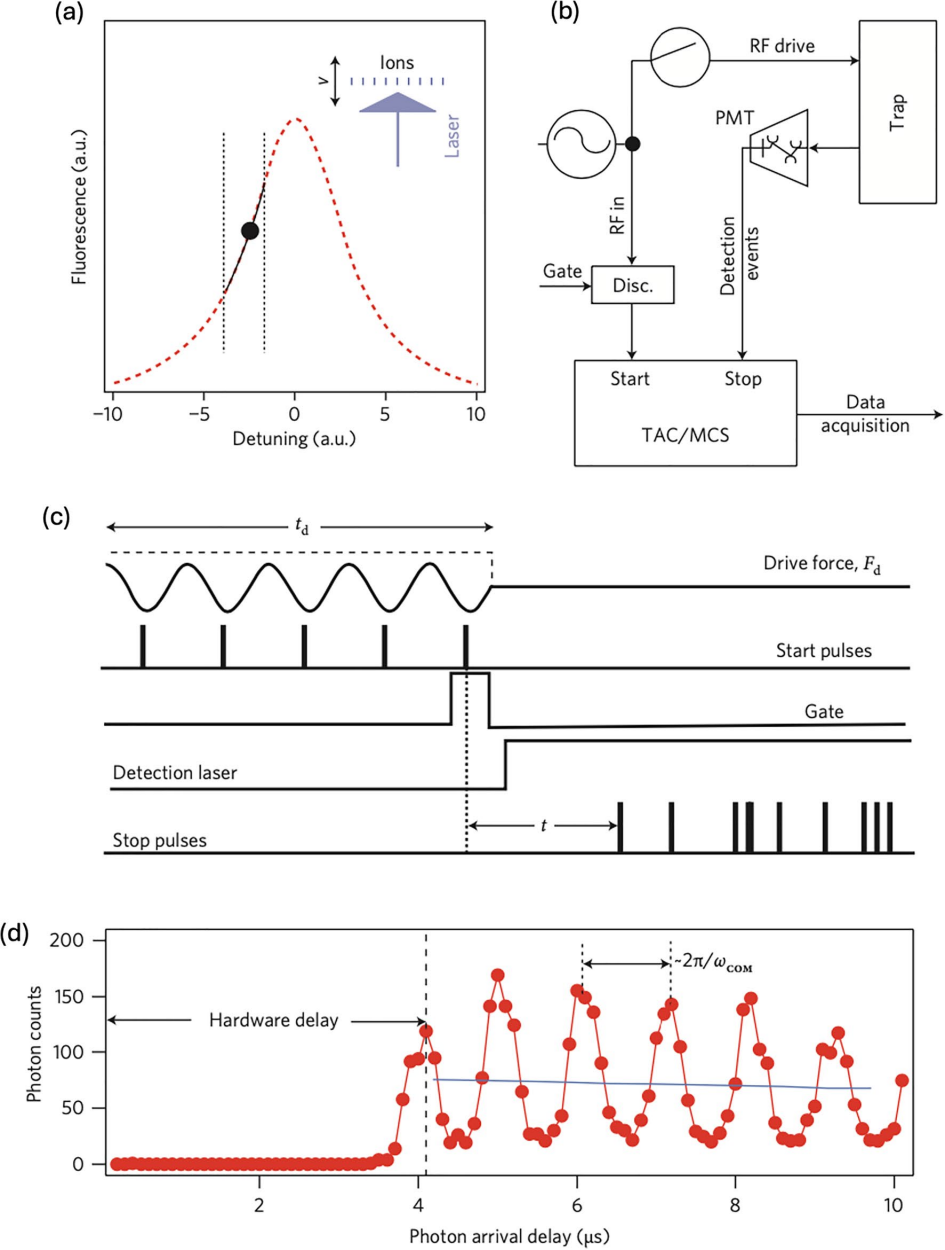
Phase-coherent Doppler velocimetry. **a** A schematic of the relationship between the fluorescence of ions and their motion. **b** The measurement protocol based on photon-arrival time measurements. **c** A schematic of pulse sequencing for Doppler velocimetry. **d** Histogram of photon arrival times relative to start pulses generated synchronously with an RF drive of the center-of-mass (COM) motional mode. [Fig. 12 reprinted with permission from ref [22], Copyright (2010) Nature Nanotechnology.]

Figure 12 indicates the measurement of the collective motion of an ion crystal with $n\approx 100$ using Doppler velocimetry. The collective motional modes of the ion crystal, such as the center-of-mass (COM) mode with a frequency referred to as ${\omega }_{z}$, are linked to the fluorescence of the crystal due to the Doppler effect. This relationship implies that the amplitude of the ion crystal’s velocity is mapped onto the photon count from the ions’ fluorescence, which is modulated at ${\omega }_{z}$ as depicted in Fig. 12a.

To test AC force sensing, an AC electric field, which is the electric force divided by the ions’ charge, is applied to one of the electrodes used for trapping the ions (Fig. 12b). This AC field exerts a force given by ${F}_{d}\text{sin}\left({\omega }_{d}t\right)$ on the ion crystal, where ${F}_{d}$ and ${\omega }_{d}$ represent the amplitude and frequency of the total force applied to the $n$-ion crystal, respectively. When the driving time is sufficiently long compared to the period of the crystal’s motion (${t}_{d}\gg 2\pi /{\omega }_{d}$), the ion crystal will oscillate with velocity ${\dot{z}}_{com}\left(t\right)=v\text{sin}\left[{\omega }_{z}t+\phi \right]$, where the amplitude $v$ and the phase $\phi$ are given by $v=\frac{2{F}_{d}{\omega }_{d}}{nm\left({\omega }_{z}^{2}-{\omega }_{d}^{2}\right)}\text{sin}\left[\frac{\left({\omega }_{d}-{\omega }_{z}\right)t}{2}\right]$ and $\phi =\frac{\left({\omega }_{d}-{\omega }_{z}\right){t}_{d} }{2}$ respectively, with $n$ being the number of ions and $m$ is the mass of a single ion. These equations are derived by calculating a harmonic oscillator under the external driving force of ${F}_{d}\text{sin}\left({\omega }_{d}t\right)$. Consequently, the velocity ${\dot{z}}_{com}\left(t\right)$ is directly correlated with the photon count from the fluorescence of the crystal, as shown in Fig. 12c and d. Since this method employs modulation at a known frequency and phase-sensitive detection, the photon count from the ion motion is solely induced by the external driving force ${F}_{d}$ and is effectively decoupled from background noise. This technique has also been primarily utilized in trapped-ion based quantum computing system to measure and compensate for the excessive micromotion of ions [22].

**Fig. 13:**
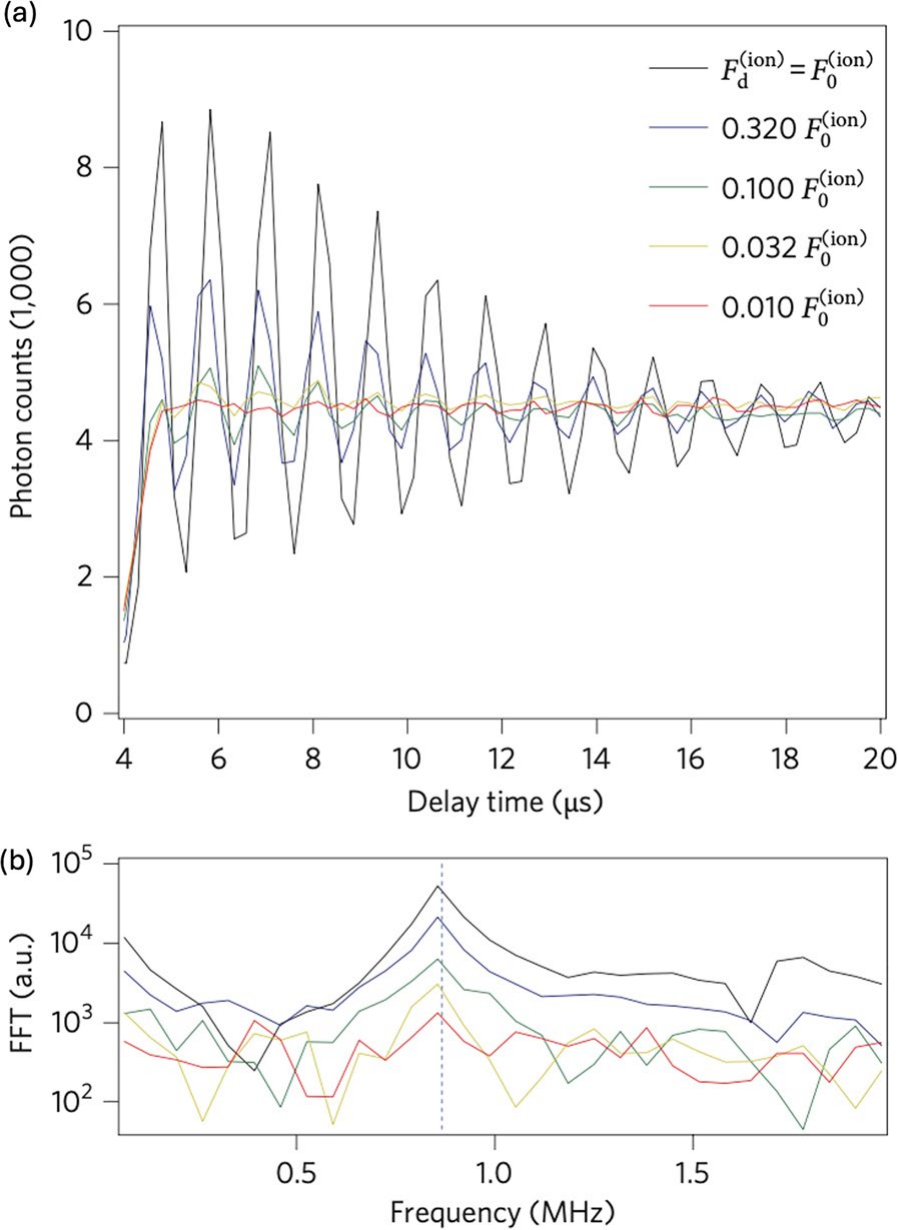
Calibration of force-detection sensitivity by Fourier analysis. **a** Temporal response to applied $F_0^{(t o t)} = n F_0^{(i o n)}$ with $n=130$ ions for decreasing electric forces and driving time $t_d = 1 \, \text{ms}$. **b** FFT of temporal response traces recorded in (**a**). [Fig. 13 reprinted with permission from ref [22], Copyright (2010) Nature Nanotechnology]

Figure 13a presents the Doppler velocimetry measurements for AC electric forces with different amplitudes $$v$$ at a constant driving frequency $${\omega }_{d}$$, which is measured in the same way as in Fig. 12d. To precisely determine the force sensitivity, it is essential to calibrate the force using a well-calibrated driving force, denoted as $${F}_{0}$$. This is achieved by applying a known radiofrequency voltage $${V}_{0}$$ to one of the trap electrodes, yielding a calibrated force $${F}_{0}$$ based on the relation $${F}_{0}=q{E}_{0}=q{V}_{0}/{d}_{0}$$, where $$q$$ is the charge of the ion, $${E}_{0}$$ is the electric field, and $${d}_{0}$$ is the distance between the trap electrode and the ion crystal. For calibration, a voltage of $$165\pm$$ 10$$\mu {\text{V}}$$ is applied, corresponding to an electric force of $${F}_{0}^{(ion)}=290\pm 18 \text{yN}$$ per ion. In the sensitivity measurements, the external driving force $${F}_{d}^{\left(ion\right)}(=$$ $${F}_{d}/n)$$ begins at $${F}_{0}^{\left(ion\right)}(={F}_{0}/n)$$ and gradually lowers to two orders of magnitude ($${0.01F}_{0}^{\left(ion\right)}$$) as shown in Fig. 13a. To find out the minimal detectable force, a Fourier transform is performed as depicted in Fig. 13b. A force of $$0.010{F}_{0}^{\left(ion\right)}$$ is detectable with a signal-to-noise ratio (SNR) of ~ 2.3 for the spectral peak, corresponding to a force per ion of approximately $$2.9 \text{yN}$$. Moreover, the collective displacement of the ion crystal under this force can be obtained by integrating $${\dot{z}}_{com}(t)$$ and reaches on the order of tens of nanometers.

12 $${z}_{com}=\frac{{F}_{d}{t}_{d}}{2nm{\omega }_{z}}\sim 18 \text{nm}$$

where $n$ is the number of trapped ions and $m$ is the atomic mass of each ion. In this Doppler velocimetry-based experiment, the collective ions’ motion functions as an ultrasensitive harmonic oscillator and enables the sensing of forces in the yocto-Newton range with a spatial resolution on the nanometer scale. This enhanced spatial resolution allows for more accurate measurements of the local environment around the ion sensors, enabling the detection of subtle changes and gradients in electric force and field.

### Squeezed state—motion coupling

Squeezed state is a quantum state that reduces the uncertainty of a specific physical variable (e.g., the position or momentum of a mechanical oscillator), while maintaining the total uncertainty of non-commuting variables, as dictated by the uncertainty principle. This implies that one could achieve more precise measurements beyond the standard quantum limit by squeezing the probability distribution of a variable (e.g., displacement), thus enhancing spatial resolution and electric/magnetic field sensitivity [23, 41, 42].

**Fig. 14:**
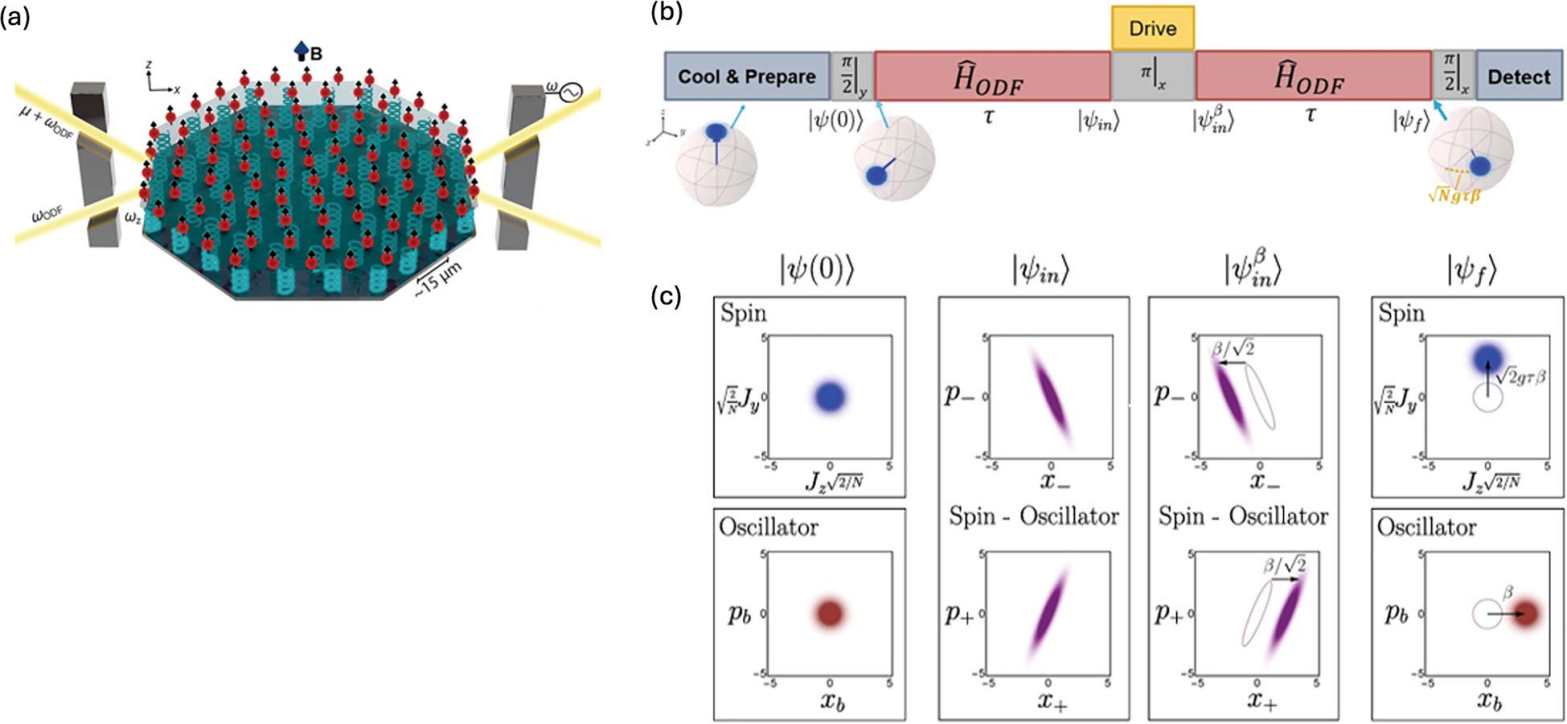
**a** A schematic describing an ensemble of trapped ${\text{Be}}^{+}$ ions in a Penning trap, **b** A schematic of displacement sensing using squeezed state, **c** A phase-space illustration after each sequence, $\hat{x}_{\pm}$ and $\hat{p}_{\pm}$ are quadrature operators of a pair of effective oscillators as follows, $\hat{x}_{\pm} = \pm (\hat{a} + \hat{a}^{\dag} \pm \sqrt{4 / N} \hat{J}_z) / 2$ and $\hat{p}_{\pm} = \pm i (\hat{a} - \hat{a}^{\dag} \pm i \sqrt{4 / N} \hat{J}_y) / 2$. [Fig. 14 reprinted with permission from ref [23], Copyright (2021) Science AAAS]

Figure 14 illustrates the scheme for displacement sensing using squeezed state with approximately 150 ${\text{Be}}^{+}$ ions ($N$ ~ 150) in a Penning trap. The ion crystals in the two-dimensional trap potential exhibit the collective center-of-mass (COM) motion (${\omega }_{z}=2\pi \times 1.59 \text{MHz}$) which behaves as mechanical oscillators (Fig. 14a).

After preparing the ions to the state$\left|\uparrow \rangle \right.$, a microwave $\pi /2$ pulse rotates the spin ensemble into superposition state $(\left|\uparrow \rangle \right.+\left|\downarrow \rangle )\right./\sqrt{2}$, denoted as$\uppsi \left(0\right)$. Then, a spin-dependent optical dipole force (ODF) which is near-resonant with the COM mode is applied for a duration $\tau$ to entangle the spins and the phonons. The system can be approximated by the Hamiltonian shown below.

13 $$\hat{H}_{{{\text{ODF}}}} = \frac{{\hbar g}}{{\sqrt N }}\left( {\hat{a} + \hat{a}^{\dag } } \right)\hat{J}_{z} - \hbar \delta \hat{a}^{\dag } \hat{a}$$

where $\hslash$ is the Planck constant divided by $2\pi$, ${\widehat{a}^{\dag}}$ and $\widehat{a}$ are the COM phonon creation and annihilation operators that couple uniformly to all spins with strength $g$, the detuning from the COM mode frequency is given by $\delta$, and ${\widehat{J}}_{z}$ are the collective spin operators. The ODF Hamiltonian (${\widehat{H}}_{\text{ODF}}$) can be approximated as a squeezing Hamiltonian under the condition of $N\gg 1$. The wave function after the first ODF is then formulated as shown below.

14 $$\left|{\uppsi }_{\text{in}}\rangle \right.=\text{exp}\left[-i\frac{\hslash g\tau }{\sqrt{N}}\left(\widehat{a}+{\widehat{a}^{\dag }}\right){\widehat{J}}_{z}\right]\left|\uppsi \left(0\right)\rangle \right.$$

When a weak electric force is applied to ion crystals, it generates a displacement $\upbeta$, which corresponds to the oscillation amplitude over time resulting from the interaction between the ions and the electric force. In this experiment, a well-calibrated AC electric field is applied to an endcap electrode along z-direction (Fig. 14a) during ${t}_{drive}\ll \tau$ to induce a small coherent displacement $\upbeta$ of the COM oscillator. As a result, the wave function becomes as shown below.

15 $$\left|{\uppsi }_{\text{in}}^{\upbeta }\rangle \right.=\text{exp}\left[\upbeta \left(\widehat{a}-{\widehat{a}^{\dag }}\right)\right]\left|{\uppsi }_{in}\rangle \right.$$

Simultaneously, a microwave $\pi$ pulse is applied to reverse the sign of the collective spin and subsequent resonant ODF is applied to optimize the largest signal, which is equivalent to opposite-sign ODF (${-\widehat{H}}_{ODF}$). This sequence drives a time-reversal step to disentangle the spins and the oscillator. In sequence, a second microwave $\pi /2$ pulse maps the displacement to accessible spin observables. Then the final wave function can be written as shown below.

16 $$\left|{\uppsi }_{\text{f}}\rangle \right.=\text{exp}\left[-i\frac{\hslash g\tau }{\sqrt{N}}\left(\widehat{a}+{\widehat{a}^{\dag }}\right){\widehat{J}}_{z}\right]\left|{\uppsi }_{\text{in}}^{\upbeta }\rangle \right.\equiv \text{exp}\left(\frac{2ig\tau\upbeta }{\sqrt{N}}{\widehat{J}}_{z}\right)\text{exp}\left[\upbeta \left(\widehat{a}-{\widehat{a}^{\dag }}\right)\right]\left|\uppsi \left(0\right)\rangle \right.$$

This pulse sequence and corresponding phase space uncertainties of spin and oscillator are indicated in Fig. 14b and c respectively. From the final state of $\left|{\uppsi }_{\text{f}}\rangle \right.$, the displacement of the COM mode is mapped onto a collective spin rotation of angle $\varphi =2g\tau\upbeta /\sqrt{N}$ by using collective spin measurement.

For displacement sensing, the signal-to-noise ratio (SNR) of a single measurement of $\upbeta$ is given as shown below, under the condition that the initial thermal occupation of the COM mode is $\overline{n }\approx 5$.

17 $$\frac{\beta }{\Delta \beta } = \frac{{2{\text{g}}\tau \beta {\text{exp}}\left( { - \Gamma \tau } \right)}}{{\sqrt {1 + \exp \left( { - 2\Gamma \tau } \right)[\left( {2\overline{n} + 1} \right)g^{2} \sigma^{2} \tau^{2} + \frac{4}{9}g^{4} \sigma^{2} \tau^{6} } }}$$

**Fig. 15:**
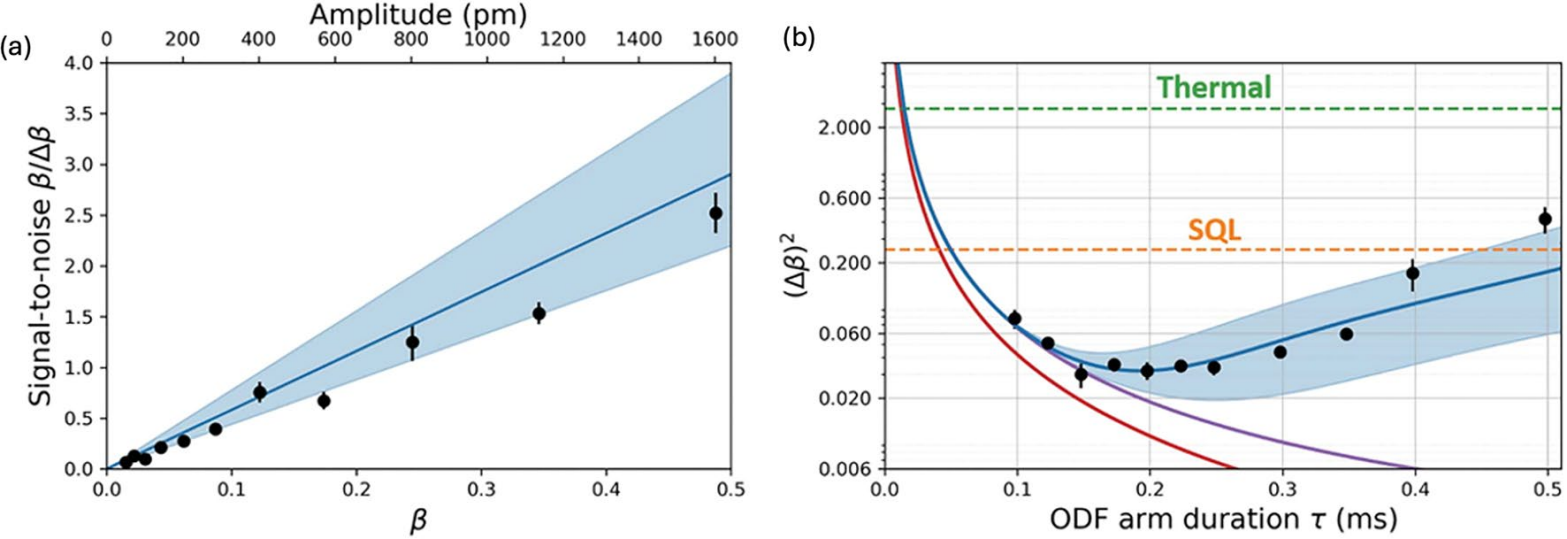
Performance of the mechanical displacement sensor. **a** Signal-to-noise ratio ($\upbeta /\Delta\upbeta$**)** as a function of displacement $\upbeta$ for a fixed ODF duration time $\tau$ of $0.2 \, \text{ms}$. The black dots represent experimental data, while the blue curve and shaded area correspond to the theoretical value and its confidence region, respectively. **b** Sensitivity **(**${\left(\Delta\upbeta \right)}^{2}$) as a function of ODF duration time at the signal-to-noise ratio of $\upbeta / \Delta \upbeta \sim 1$ from (**a**). The black dots, blue curve, and shaded area indicate experimental data, theoretical value, and its confidence region, respectively. The red and purple curves depict theoretical predictions for an idealized model and a model that accounts for depolarization due to spin decoherence, respectively. The dashed lines indicate the thermal noise limit (green) and the standard quantum limit (orange). [Fig. 15 reprinted with permission from ref [23], Copyright (2021) Science AAAS]

Here, $\sigma$, $\Gamma$ represent the frequency fluctuations of the COM mode with a root-mean-square spread and the single-particle decoherence rate of the spins due to light scattering generated by the applied ODF pulse, respectively. This result can be reformulated as the absolute physical displacement ${\text{Z}}_{c}$ of the COM mode, $\upbeta /\Delta\upbeta \equiv {\text{Z}}_{\text{c}}/\Delta {\text{Z}}_{\text{c}}$. The amplitude of COM mode, ${\text{Z}}_{\text{c}}$ can be written as ${\text{Z}}_{\text{c}}=2{\text{z}}_{0}\upbeta /\sqrt{N}$ where ${\text{z}}_{0}=\sqrt{\hslash /(2m{\omega }_{z})}$ and $m$ is the atomic mass of each ion. Figure 15a displays the signal-to-noise ratio ($\upbeta /\Delta\upbeta$) as a function of displacement ($\upbeta )$ at a fixed ODF duration time $\tau =0.2 \text{ms}$. When the signal-to-noise ratio ($\upbeta /\Delta\upbeta$ ) becomes approximately one, $\upbeta$ and ${\text{Z}}_{\text{c}}$ correspond to 0.24 and $775\pm 28 \text{pm}$, respectively. For a fixed $\upbeta$ of 0.24, the sensitivity (${\left(\Delta\upbeta \right)}^{2}$ ) can be measured as a function of the duration time $\tau$ as shown in Fig. 15b. The optimal sensitivity is achieved at $\tau \approx 0.2 \text{ms}$, where the value of ${\left(\Delta\upbeta \right)}^{2}=0.033$, which is $8.8\pm 0.4 \text{dB}$ below the standard quantum limit (SQL) of ${\left(\Delta\upbeta \right)}^{2}=0.25$.

**Fig. 16:**
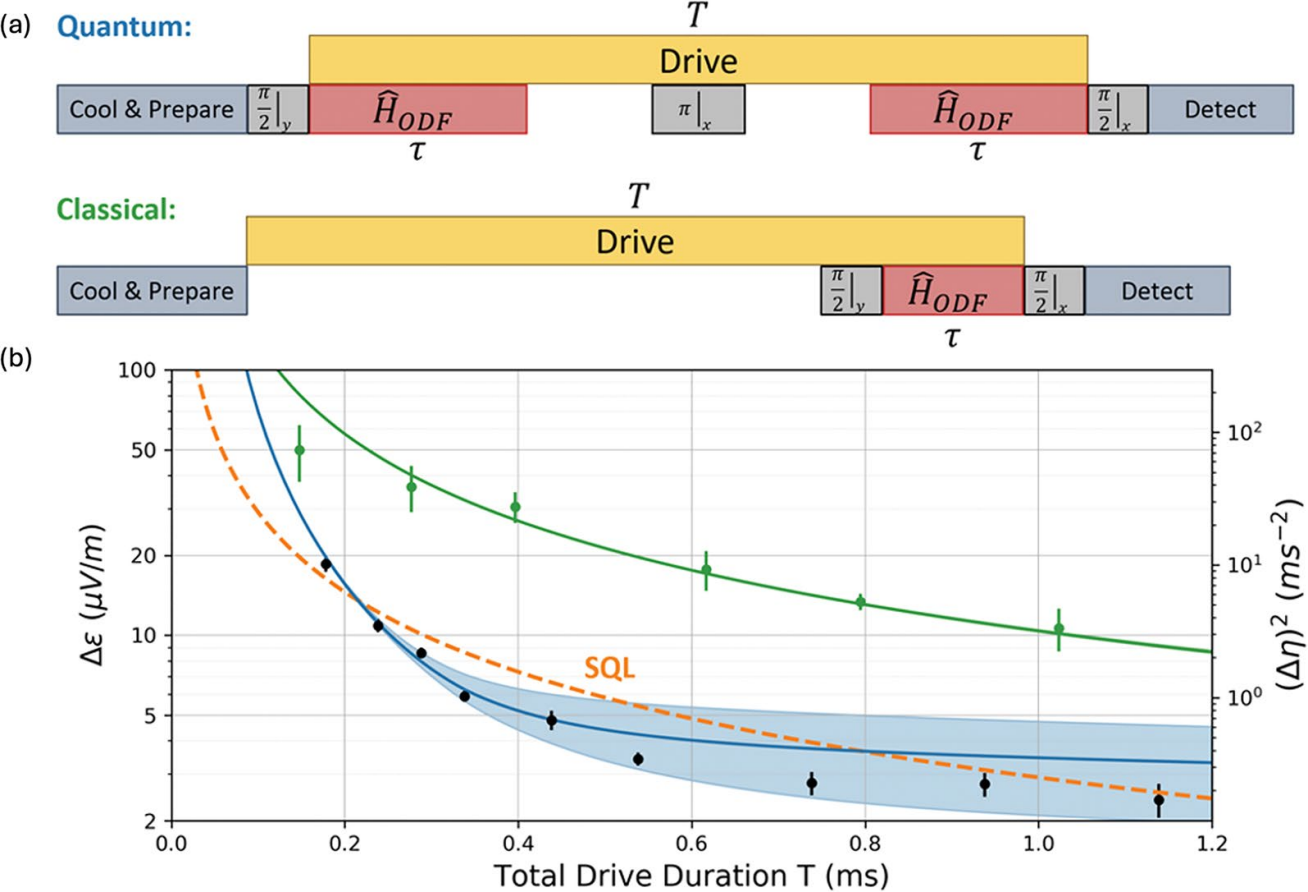
Performance of the electric field sensor. **a** Procedures for both the quantum and classical approaches to measure weak electric fields. **b** Electric field sensitivity (∆ε) as a function of the total drive duration ${\varvec{T}}$. Green and black dots correspond to experimental results from both classical and quantum protocol, respectively. Green and blue lines indicate the theoretical models of both protocols, and shaded area represents the confidence region. Standard quantum limit is depicted by orange dashed line. [Fig. 16 reprinted with permission from ref [23], Copyright (2021) Science AAAS]

The use of squeezed state can also probe weak AC electric fields by measuring the small displacement of the COM mode, as described above. Figure 16a illustrates the procedures for both the quantum and classical approaches to measure the electric fields. In this context, the classical approach refers to a conventional detection scheme while the quantum approach employs squeezed states for displacement detection, as described above. When a fixed AC electric field is applied to the ion crystal for a total time $T$, the first ODF pulse generates spin-motion entanglement for a time $\tau \le T/2$. At a subsequent time, a microwave $\pi$ pulse flips the sign of the collective spin, and a second ODF pulse follows for a duration $\tau$ (which serves as a time-reversal operation for the first ODF pulse). The final state is then detected after a second microwave $\pi /2$ pulse. The Hamiltonian for this system includes a perturbation term due to the applied electric field and is expressed as:

18 $${\widehat{H}}_{\text{sens}}={\widehat{H}}_{\text{ODF}}\left(t\right)+i\eta \left(\widehat{a}-{\widehat{a}^{\dag}}\right)$$

where $\eta$ is a parameter related to the electric field being measured. By solving for the final wavefunction $\left|{\uppsi }_{\text{f}}\rangle \right.$ based on the ${\widehat{H}}_{\text{sens}}$, one can calculate the sensitivity ${\left(\Delta \eta \right)}^{2}$ of the system. In the classical approach, only a single ODF pulse is used to detect the continuous displacement of the COM mode. This classical protocol results in a sensitivity ${\left(\Delta \eta \right)}_{c}^{2}=\left(1+{g}^{2}{\tau }^{2}\right)/[{g}^{2} {\tau }^{2}{\left(2T-\tau \right)}^{2}]$. Here, the total displacement of the oscillator is given by $\upbeta \equiv \eta T$. For the quantum method, quantum-enhanced sensitivity is described by ${\left(\Delta \eta \right)}^{2}=1/[4{g}^{2} {\tau }^{2}{\left(T-\tau \right)}^{2}]$ which can be approximated as $4/({g}^{2} {T}^{4})$ under the condition of $g\tau =gT/2\gg 1$. Figure 16b presents the electric field sensitivity ($\Delta \varepsilon$ and ${\left(\Delta \eta \right)}^{2}$) as a function of total drive duration $T$. Here, $\Delta \varepsilon$ is the standard deviation of a single measurement of the electric field. The quantum protocol achieves a sensitivity of $\sim 4.0\pm 0.5\text{dB}$ below the standard quantum limit for a driving time $T = 538\mu {\text{s,}}$ and also improves the sensitivity by approximately $14 \text{dB}$ compared to the classical protocol. For long drive times ($T\ge 1\text{ms}$), the optimal electric field sensitivity is obtained by $\Delta E=\Delta \varepsilon \sqrt{{T}_{\text{shot}}}=220\pm 10{\text{nVm}}^{-1}/\sqrt{\text{Hz}}$ at the total duration of the experimental trial, ${T}_{\text{shot}}=8.73 \text{ms}$.This result demonstrates that the trapped-ion system can utilize squeezed states to reduce uncertainty in displacement and electric field measurements. Squeezed states, known for their ability to lower quantum uncertainties, enable sensing these quantities with greater precision, down to the nanometer scale for displacement and nanovolt ($\text{nV}$) level for electric fields. This highlights the potential of the trapped-ion system for advancing high-precision sensing applications.

## Conclusion and perspectives

With advancements in quantum technologies, achieving precise and accurate measurements of physical quantities using quantum phenomena has been actively pursued toward reaching and even overcoming the standard quantum limit, surpassing the inherent limitations of classical techniques. Among the various types of quantum sensors, trapped-ion based sensors are particularly promising, as they leverage the atomic states provided by nature, offering high precision and accuracy. Furthermore, the long coherence time of trapped-ion qubits allows for enhanced sensitivity and the trapped-ion qubits enable spatial resolution beyond the diffraction limit of light through advanced measurement techniques, reaching even the nanometer scale.

From the perspective of quantum sensing, techniques for qubit phase accumulation, extending coherence time, minimizing noise from the local environment, and various modulation and detection methods have been developed to achieve unprecedented sensitivity for electric and magnetic fields, alongside improved spatial resolution. This review introduces key experimental methodologies using trapped-ion sensors, including dynamic decoupling, dressed states, quantum entangled states, ultra-high-resolution techniques, Doppler velocimetry, and quantum squeezed states.

Future developments in trapped-ion based quantum sensors may focus on multi-qubit control and sensing, enhancing magnetic and electric field sensitivity with remote sensing capabilities through multi-qubit entanglements. For instance, a group of large-scale entangled ion qubits could be shuttled around a region of interest for sensing, enabling robust quantum coherence and better sensitivity through increased ion qubits. This approach would also allow for precise spatial field distribution and gradient measurement. Moreover, integrating scanning capabilities into the trapped-ion system to use the ions as scanning probes may offer an intriguing research avenue. This would replace classical scanning probe tips or cantilevers with a quantum object—ion qubit—which could act as a pristine quantum sensor. Such a system could facilitate the probing of real magnetic and electric samples with enhanced sensitivity and spatial resolution.

For several decades, the trapped-ion system has been extensively investigated for quantum metrology and quantum computing. In recent years, its applications have expanded to include quantum communication and sensing. In this review, we have introduced various quantum sensing methodologies alongside experimental advancements in the trapped-ion system, and we believe that continued progress will enable quantum sensing of a wide range of physical quantities, surpassing the standard quantum limits in the future.

## Data Availability

Not applicable.
